# Biological Action Identification Does Not Require Early Visual Input for Development

**DOI:** 10.1523/ENEURO.0534-19.2020

**Published:** 2020-10-27

**Authors:** Siddhart S. Rajendran, Davide Bottari, Idris Shareef, Kabilan Pitchaimuthu, Suddha Sourav, Nikolaus F. Troje, Ramesh Kekunnaya, Brigitte Röder

**Affiliations:** 1 Biological Psychology and Neuropsychology, University of Hamburg, Hamburg, Germany; 2 Jasti V Ramanamma Children’s Eye Care Center, LV Prasad Eye Institute, Hyderabad, India; 3 IMT School for Advanced Studies Lucca, Lucca, Italy; 4 Department of Biology, Centre for Vision Research, York University, Toronto, Ontario, Canada

**Keywords:** biological action, biological motion, congenital cataract, global motion, visual deprivation

## Abstract

Visual input during the first years of life is vital for the development of numerous visual functions. While normal development of global motion perception seems to require visual input during an early sensitive period, the detection of biological motion (BM) does not seem to do so. A more complex form of BM processing is the identification of human actions. Here, we tested whether identification rather than detection of BM is experience dependent. A group of human participants who had been treated for congenital cataracts (CC; of up to 18 years in duration, CC group) had to identify ten actions performed by human line figures. In addition, they performed a coherent motion (CM) detection task, which required identifying the direction of CM amid the movement of random dots. As controls, developmental cataract (DC) reversal individuals (DC group) who had undergone the same surgical treatment as CC group were included. Moreover, normally sighted controls were tested both with vision blurred to match the visual acuity (VA) of CC individuals [vision matched (VM) group] and with full sight [sighted control (SC) group]. The CC group identified biological actions with an extraordinary high accuracy (on average ∼85% correct) and was indistinguishable from the VM control group. By contrast, CM processing impairments of the CC group persisted even after controlling for VA. These results in the same individuals demonstrate an impressive resilience of BM processing to aberrant early visual experience and at the same time a sensitive period for the development of CM processing.

## Significance Statement

Biological motion (BM) is a crucial aspect of human vision, which has been shown to emerge early in human ontogeny. Here, we report an astonishing high accuracy in identifying human actions in a unique group of individuals who had regained vision later in life (until the age of 18 years) after being treated for congenital cataracts (CC). By contrast the same individuals were markedly impaired in another non-BM tasks requiring the detection of motion coherence in dot kinematograms, even after visual acuity (VA) impairments were taken into account. Thus, the present study demonstrates a remarkable resilience of complex BM processing capabilities such as the identification of human actions to aberrant early visual experience.

## Introduction

Sensory input during early years of life is essential for normal development of sensory systems (Wiesel and Hubel, 1965). In humans, studies in individuals treated for congenital cataracts (CC) have revealed incomplete recovery in many visual functions, including visual acuity (VA; Ellemberg et al., 1999), stereovision (Tytla et al., 1993), visual feature binding (Putzar et al., 2007; McKyton et al., 2015), global motion processing (Ellemberg et al., 2002; Bottari et al., 2018), and face processing (Le Grand et al., 2001; Röder et al., 2013), while functions such as color discrimination (Brenner et al., 1990; Pitchaimuthu et al., 2019) seemed to emerge independently of early visual experience. Biological motion (BM) processing, e.g., the ability to detect the movement of biological figures with sparse information (Johansson, 1973), has been shown to recover well following early visual deprivation (Hadad et al., 2012), that is, both detection thresholds as well as neural signatures have been observed to be indistinguishable between a CC group and normally sighted controls (SC group; Bottari et al., 2015, 2016).

However, it is unclear yet whether more complex aspects of BM processing, such as the identification of human actions, require early visual experience. BM detection, as studied with point light displays of walkers, requires the extraction of the spatial configuration of point lights and its congruent change over time (Theusner et al., 2014). In contrast, action identification involves knowledge of the meaning of human postures and how they change over time. Action identification is further complicated by the large variance from different agents performing the actions and the different viewpoints from which actions are observed. Action identification, thus, requires extracting action invariant features.

In the present study, we tested individuals with a history of pattern vision loss because of bilateral, total, dense CC on their ability to accurately perceive human actions performed by line figures. For this purpose, we used the test battery (BMLtest battery) developed by Saunders and Troje (2011). Three potential confounds were additionally investigated: first, unspecific effects related to cataract surgery; second, the timing of visual deprivation, that is, whether the visual deprivation existed at birth or emerged later in development; third, the overall lower VA typical for individuals with a history of early cataracts. In previous studies, both of these confounds have been simultaneously controlled for by including individuals who had lower VA compared with typically sighted individuals because of cataract onset later during development [e.g., individuals with reversed developmental cataract (DC); Lewis and Maurer, 2009; Röder et al., 2013]. Alternatively, the persisting visual impairments have been controlled for by blurring visual stimuli for normally SCs (McKyton et al., 2015). If a sensitive period exists for the development of a specific function, once VA is controlled for, the CC group, but not the DC group is expected to show impaired performance. The normally sighted age matched controls were tested with full sight and with the VA matched using specialized translucent filters called Bangerter filters, which have been regularly used in amblyopia therapy (Agervi, 2011). All participants were tested in two tasks of the BMLtest battery (Saunders and Troje, 2011): (1) an action identification task, in which subjects had to name the action performed by an actor shown as stick figures; (2) a coherent motion (CM) detection task, in which participants judged the direction of the CM of dots among a set of randomly moving dots (CM task).

We predicted that complex aspects of BM, such as the identification of human actions, would depend less on early visual experience than CM perception. Thus, we hypothesized that any group difference between the CC group and SC group in the action identification task can be accounted for by VA differences. By contrast, for the CM task we predicted that impairments specific for the same CC group emerge even after controlling for VA. Thus, the present study is aimed at providing non-confounded evidence for a sensitive phase in early development for global motion processing while showing in the same individuals a high resilience of complex BM functions as the identification of human actions to aberrant early visual experience.

## Materials and Methods

### Participants

The group of individuals with a transient phase of CC (CC group) was comprised of 12 participants (mean age: 16 years, range: 7–34 years; three females; all right handed; mean age at surgery: 66.8 months, range: 2–220 months; mean duration since surgery: 120 months, range: 6–396 months; for detailed demographics of participants, refer to Table 1) who had undergone treatment for bilateral, dense, CC in both eyes. These participants were recruited from LV Prasad Eye Institute, Hyderabad, India. Congenital cataract individuals qualifying for the present study were identified among a larger number of patients with the diagnosis “congenital cataract” based on the following criteria. (1) All patients suffered nystagmus which is often the result of a lack of pattern vision for longer than 10–13 weeks after birth (Rogers et al., 1981; Gelbart et al., 1982). Such a nystagmus persists after surgery, and was present in all CC individuals. (2) The lenticular opacity for CC individuals was dense before surgery which resulted in an inability to have a view of the fundus during clinical examination (except participants with partially absorbed cataracts). Additionally, (3) a positive family history of congenital cataract. (4) Esotropia resulting from equal visual deprivation in both eyes was used as additional classification criteria. This information was available from the detailed medical records available at LV Prasad Eye Institute, Hyderabad, India. Patients with an ambiguous clinical profile were not included. Although a 100% confidence of having included only congenital cataract-reversal individuals with a history of bilateral, total, dense cataracts is not possible in a retrospective classification of participants, the likelihood of achieving an accurate CC classification was maximized in the present sample by having access to extensive clinical data allowing for the use of strict criteria.

**Table 1:** Clinical information of the congenital cataract reversal group (CC group) and age/sex matching with the VM control group

Participant	Strabismus	Family history	VA at surgery (better eye)	Dense	Nystag- mus	Age at surgery (months)	Duration since surgery (months)	Age at test (years)	VA at test (logMAR)	Sex #	Age VM (years)	VA VM (logMAR)	Sex VM #
CC1	Exotropia	No	Fixates light	Yes	Yes	4.5	103.5	9	0.48	M	9	0.53	M
CC2	Exotropia	Yes	Fixates and follows light	Yes	Yes	5	139	12	0.48	M	12	0.5	M
CC3	Esotropia	Yes	Fixates and follows light	Yes	Yes	2	106	9	0.3	F	9	0.39	F
CC4	Exotropia	Yes	Finger counting at 0.5 m	Yes	Yes	165	27	16	0.9	F	16	0.91	F
CC5	Esotropia	Yes	NA	Yes	Yes	24	396	34	0.3	M	32	0.37	F
CC6	No	Yes	NA	Yes	Yes	72	348	30	1.3	M	29	1.38	F
CC7	Esotropia	No	PL/PR accurate	Yes	Yes	121	11	11	0.8	F	11	0.85	F
CC8	No	Yes	HM+	Yes	Yes	86	34	10	0.5	M	11	0.55	M
CC9	No	No	PL/PR accurate	Yes	Yes	220	6	18	1.08	M	19	0.9	F
CC10	No	Yes	1.77 logMAR	Yes	Yes	74	22	7	1.1	M	8	1.2	M
CC11	Esotropia	Yes	NA	Yes	Yes	4	236	20	1	M	19	1.09	M
CC12^*^	No	Yes	Finger counting at 3 m	Yes	Yes	84	12	8	0.8	M	9	0.9	M

*Subject with partially absorbed cataract; abbreviations used in VA columns: NA, not available in records; PL, perception of light; PR, perception of the direction of light rays; HM, hand movement close to face; #: M, male; F, female.

In order to ensure that any observed difference between congenital cataract-reversal individuals and normally SCs was not the result of trivial causes such as the experience of a surgical procedure at the eyes, and to understand the effects of vision loss immediately after birth versus later in childhood, we tested a group of control participants whose form vision was preserved at birth and who developed cataracts later during childhood. These participants had undergone the same surgical procedure as the CC group; three out of these 12 individuals had congenital but non-dense cataracts. These participants were subsumed as DC group. Similar to the CC group, these participants had undergone treatment for cataract (mean age at surgery: 116 months, range: 24–434 months; mean time since surgery: 37 months, range: 2–183 months). We tested 12 DC participants (mean age: 12.6 years, range: 8–37 years, four females, all right-handed) who were recruited from LV Prasad Eye Institute, Hyderabad, India. They were classified based on the medical records of LV Prasad Eye Institute, India. The clinical diagnosis of DC was mostly based on a number of criteria such as the parents’ or patients’ report of the age of poor vision onset, lack of nystagmus and a lack of family history of congenital eye pathologies. Additionally, individuals with incomplete congenital cataract, suggesting preservation of early pattern vision were included in the DC group (for complete description, see Table 2).

**Table 2:** Clinical information of the DC group

Participant	Strabismus	Family history	VA at surgery (better eye)	Dense	Nystag- mus	Age at surgery (months)	Duration since surgery (months)	Age at test (years)	VA at test (logMAR)	Sex #
DC1^§^	NA	NA	NA	No	NA	59	45	8	0.7	M
DC2	NA	NA	NA	NA	NA	102	43	12	0.18	M
DC3	Exotropia	No	fixates and follows objects	No	Yes	24	183	17	0.18	F
DC4	NA	NA	NA	NA	NA	120	20	11	0.18	F
DC5	NA	NA	NA	NA	NA	73	48	10	0.18	F
DC6	NA	NA	NA	No	NA	83	44	10	0.4	M
DC7^§^	NA	Yes	20/200	No	Yes	434	9	36	1	M
DC8	No	No	PL+ PR accurate	Yes	Yes	110	10	10	1.7	M
DC9	No	No	20/100	No	No	97	6	8	0	M
DC10	NA	No	20/80	No	No	84	11	7	0.3	M
DC11	Exotropia	No	CF 2 m	No	No	96	25	10	0	F
DC12^§^	Exotropia	No	CF 1 m	No	Yes	105	2	8	1.3	M

§ Individuals with incomplete congenital cataract; abbreviations used in VA columns: NA, not available in records; PL, perception of light; PR, perception of the direction of light rays; HM, hand movement close to face; #: M, male; F, female.

To control for VA and thus to isolate the effects of prevailing VA loss at the time of testing, a second control group of 12 participants with normal vision (mean age 15.3 years, range: 8–32 years; 6 females; all right-handed; for the exact VAs, refer to Table 1) was tested. Similar to the CC and DC participants, these individuals were also recruited from Hyderabad, India. They were matched for age with the CC group (CC; ±1 year, for one adult CC participant, 2 years, see Table 1). This control group performed the task twice: once with normal vision and the second time with the VA being individually matched to one of the CC individuals. With their vision reduced using the Bangerter filters, these participants served as the vision matched (VM) control group, and without the filters the same participants served as normally SCs. Hence, with their reduced vision, the control group was matched for age and VA with that of the CC group.

Participants in all three groups (CC, DC, VM/SC) were healthy (expect the history of cataracts in CC and DC individuals) with no history of physical problems by self-report (VM/SC group) and by physical examination (CC and DC groups). The study was approved by the institutional ethics boards of University of Hamburg, Hamburg, Germany and LV Prasad Eye Institute, Hyderabad, India. Informed consent was obtained from participants, and from legal guardians for minors before the beginning of experiments. The quality of health care received by the participants was not affected by their willingness to participate in our experiments.

### Stimuli and apparatus

#### VA matching using Bangerter filters

Bangerter filters are translucent light diffusers that can be attached to spectacles and are capable of causing degradation in vision because of the resultant attenuation of higher spatial frequencies (Pérez et al., 2010). Although these filters can reduce the VA in steps on 0.1 logMAR in healthy eyes (Odell et al., 2008; Agervi, 2011; Rutstein et al., 2011), an intended degradation of >1.0 logMAR of VA cannot be obtained even by the manufacturer’s recommendation. Since our participants required VA reduction of up to 1.3 logMAR, we used a combination of filters to achieve VA reductions beyond 1.0 logMAR. For this purpose, we attached the filters to zero power wide field trial lenses typically used in optometric eye examinations. Similar to the use of trial lenses during an eye examination to arrive at the correct refractive correction, combinations of these “trial filters” were in turn used to arrive at the desired VA. Using the manufacturer’s recommendations and previous reports (Odell et al., 2008) as starting filter strengths, different combinations of filters were tried until the desired VA was obtained. Although not tested with Bangerter filters, the neural system is generally prone to blur adaptation (Clifford et al., 2007; Webster, 2015), and hence, the participants were asked to stay with the filters for a few minutes for the visual system to get adapted to the blur, and VA estimation was repeated after adaptation. If the pre and post VA varied widely, the filter strength was assessed again until a stable filter strength was obtained. While combining multiple filters, the “microbubbles” (Pérez et al., 2010) of a given filter aligning with that of the preceding/following one might potentially be an important factor in maintaining image degradation. But in our experience, we noticed that the alignment was robust to small variations in head movements. All VA measurements were done using the Landodt’s C optotype of the Freiburg Visual Acuity Test (FrACT; Bach, 1996). The optotypes were shown on a 20’’ Dell monitor with a resolution of 1600 × 900 (refresh rate 60 Hz) and at a testing distance of 240 cm controlled by a Dell laptop.

#### Stimuli

The biomotion test battery (BMLtest) by Saunders and Troje (2011) was used. The test battery consists of a number of tests, of which we used the “coherence” and “action” experiments. All stimuli were shown on a 20’’ Dell IN2030M LCD monitor with a resolution of 1600 × 900 and a refresh rate of 60 Hz using a Dell laptop. Participants were seated at a distance of 60 cm from the screen, and viewed the stimuli with their best refractive correction. They were allowed to switch between the segments of their bifocals (in case of the CC and DC participants) and were additionally allowed to get a closer look at the stimuli in cases where the stimuli were not sufficiently visible at the testing distance. For the VM participants the Bangerter filters were placed over their refractive correction.

### Procedure

#### CM task

The “coherency test” of BMLtest battery, originally based on Newsome and Paré (1988), and used by Bottari et al. (2018) was adapted to obtain global motion coherence thresholds (CM task). In a 10° circular field, 15 dots, each of 15 pixel size moved either randomly or were displaced by 0.15° in a particular direction (right or left; the direction of motion was randomized on each trial). Each trial lasted 1000 ms and was preceded by a black screen with white fixation cross for the same amount of time. After each trial, participants gave a verbal response indicating the direction of motion (toward left or toward right). Younger children waved the appropriate hand to indicate the direction instead of providing an oral response. In all cases, the experimenter pressed the response button. The next trial did not start until participants had given a response for the current trial. The test used a QUEST staircase procedure to obtain the threshold for detecting the direction of motion with a likelihood of 82%.

#### Biological action identification task

**Figure 1.**
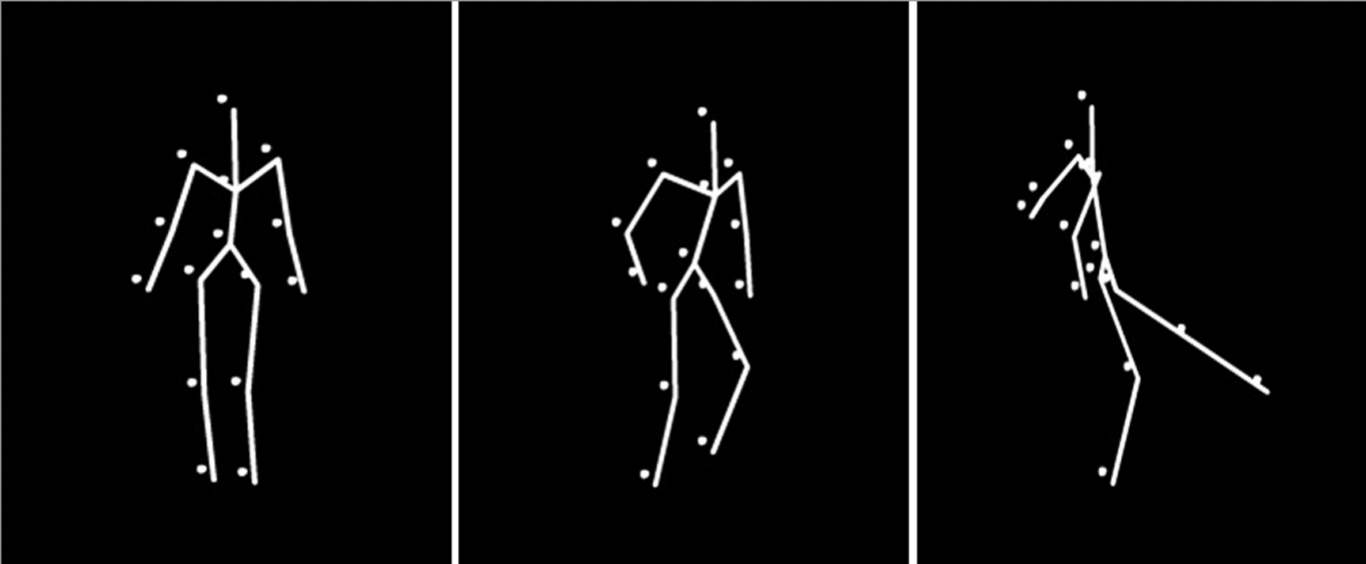
Still frames from three actions of the biological action identification task. The three actions are walking, climbing, and kicking (left to right).

The action subtest of the BMLtest battery was used to assess participants’ ability to accurately recognize an action performed by a moving line figure (see Fig. 1). The suite consisted of 10 different actions presented from three different viewpoints: straight ahead (0° view angle), oblique (30/45° view angle), and profile (90° view angle), totaling to 30 trials. The line figure was formed by connecting 14 dots located at different joints and extremities of a typical human body (see Fig. 1). Each dot of the line figure was 15 pixels in size with the full figure being 280 pixels in size. The order of these trials was randomized on each participant. Participants were made aware of the 10 possible actions before the experiment, but were encouraged to respond intuitively. The 10 possible actions presented were catching, climbing stairs, jumping, jumping jacks, kicking, lifting, running, sitting, throwing, and walking. If participants, particularly children, did not have a word to describe the action performed by the BM figure, they were asked to imitate the action. On a number of occasions, participants who found it hard to give a verbal response for an action were able to recognize the action by imitating it, which was still taken as correct response. Participants reported the action verbally in Hindi, Telugu, or English, and the experimenter pressed the response button. Reaction times were not recorded.

The CC and DC group performed the CM and biological action identification task in a random order once. The normally SCs performed both tasks twice, with (VM control group) and without (SC group) the Bangerter filters; again, the order was randomized.

### Data analysis

VA as measured using FrACT was recorded in logMAR units and the ability to detect CM was measured as the percentage of coherently moving dots needed to achieve 82% correct responses. The action task consisted of 10 actions presented from three different view angles. The number of correct responses obtained out of 30 was used to calculate the proportion of correct responses for each participant. VA (logMAR units), biological action identification (proportion of correct responses) and CM detection (threshold) were taken as dependent variables for separate group comparisons (CC, DC, VM/SC). One-way ANOVA was used to compare the CC group with the DC and VM group. A separate analysis was run comparing the CC group with the DC and the SC group. Planned comparisons between SC and VM groups were run using paired *t* test. Homogeneity of data were tested using Levene’s test, and when violated, the ANOVA was conducted assuming unequal variance.

The effect of viewpoint on the proportion of correct responses was additionally added as a repeated measurement factor in a two-way ANOVA with CC versus VM as group factor. The number of correct responses was averaged for all the 10 trials from a given viewpoint and the average score was taken as the proportion of correct responses for this view angle. All analyses were done with RStudio (RStudio Team, 2016) using appropriate packages. An effect was considered significant if the resulting *p* value for the statistical test was <0.05.

## Result

### VA comparison between groups

**Figure 2.**
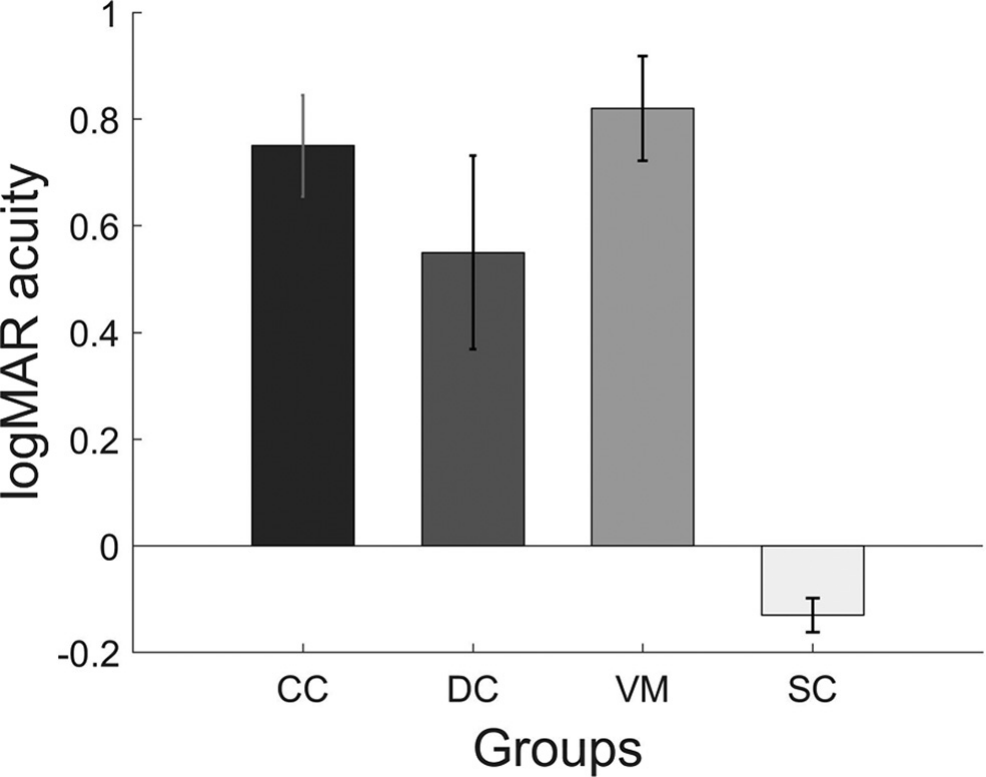
Bar plots displaying the visual acuities of each of group. Shown are the mean VA and the error bars indicate the SEM.

A one-way ANOVA comparing the mean VA between the CC, DC, and VM groups was not significant (*F*_(2,33)_ = 1.11; *p* = 0.34). However, the comparison of the mean VA between CC, DC, and SC groups using a one-way ANOVA was found to be significant (*F*_(2,16.45)_ = 41.2; *p* < 0.001). *Post hoc* pairwise *t* tests using Bonferroni corrections showed that the VA score for the SC group (mean logMAR VA = −0.13, SD = 0.11) was significantly higher than the VA scores of both the CC (mean logMAR VA = 0.753, SD = 0.33; *p* < 0.001) and the DC (mean logMAR VA = 0.556, SD = 0.63; *p *=* *0.001) group. Further, a within group comparison using paired *t* test demonstrated the successful match of the Bangerter filters: The SC group had significantly better VA (*t*_(11)_ = −10.13; *p* < 0.001), than the VM group (mean logMAR VA = 0.82, SD = 0.34; Fig. 2).

### Biological action identification task: comparison between groups

**Figure 3.**
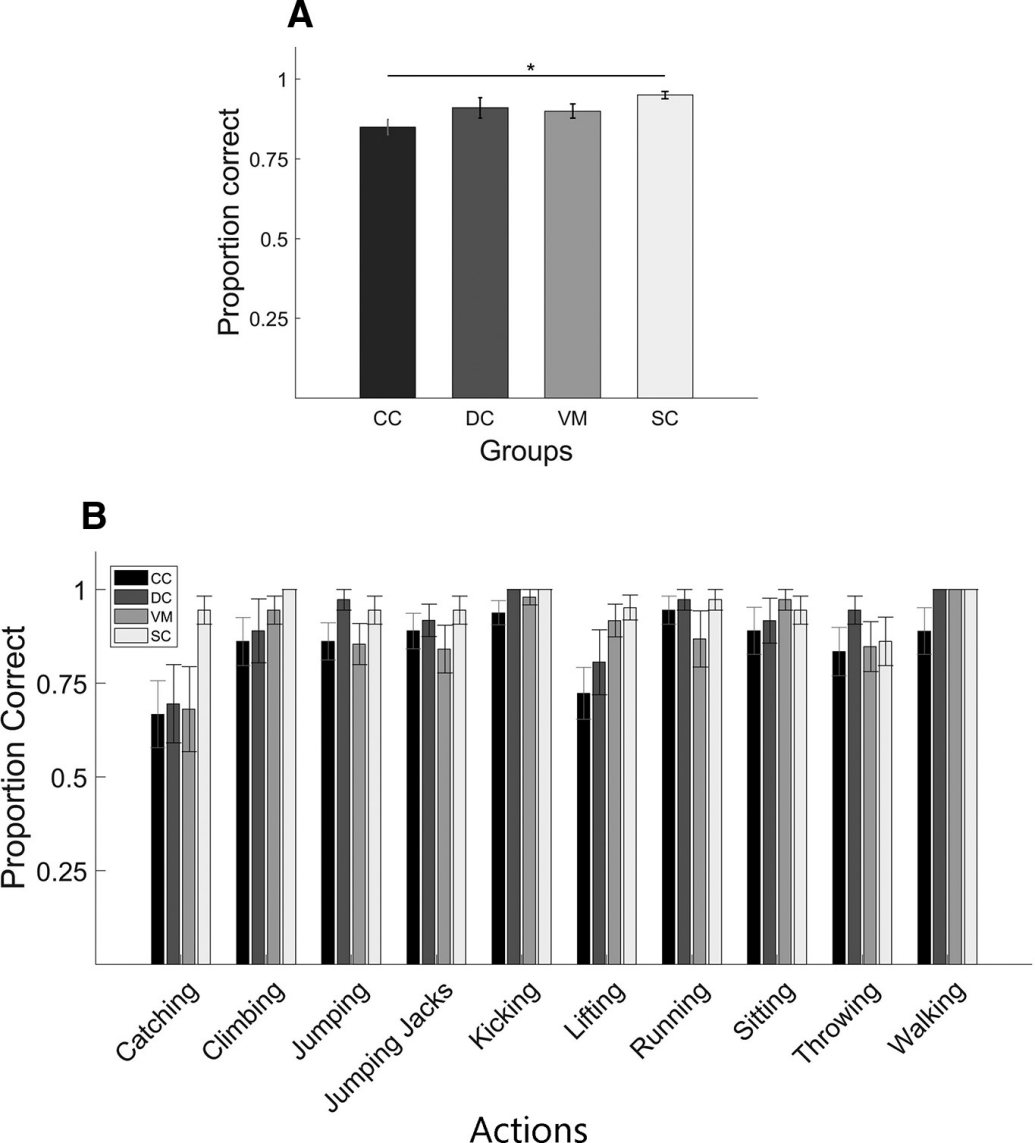
Results of the biological action identification task. ***A***, Bar plots display the proportion of correct responses for each group: CC, DC, VM, SC (for definition, see Fig. 1). Asterisk indicates significant group difference. ***B***, Same as ***A*** but proportion correct responses separately averaged for each of the 10 actions. Shown are group mean scores; error bars indicate SEM.

The one-way ANOVA with participant groups CC, DC, VM as between groups factor and proportion of correct responses as dependent variable found no significant effect of group (*F*_(2,33)_ = 1.44; *p* = 0.24), whereas the analogous ANOVA with the participant groups CC, DC, and SC groups was found to be significant (*F*_(2,33)_ = 4.34; *p* = 0.02). *Post hoc* pairwise *t* tests using Bonferroni corrections for the latter showed that the CC group (mean score = 0.85, SD = 0.08) performed significantly worse in the task than the SC group (mean score = 0.95, SD = 0.04; *p* = 0.019), but the performance of CC group was not significantly different from the DC group (mean score = 0.91, SD = 0.11; *p* = 0.25; Fig. 3*A*). As seen in Figure 3*B*, performance varied across action type but the CC group achieved a mean accuracy of 66.7% (SD = 0.3) for the action they were least able to identify.

### Effect of viewpoint on biological action identification

**Figure 4.**
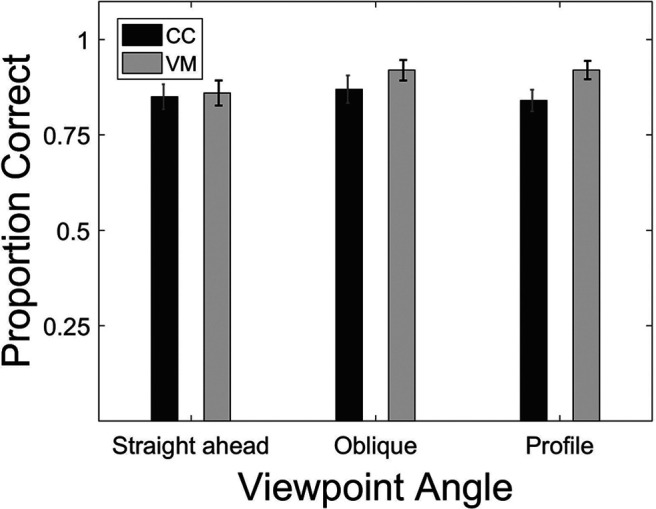
Biological action identification task. Bar plots show the proportion of correct responses separately averaged for the three viewpoints and CC and the VM participants (for definitions, see Fig. 1). Error bars indicate SEM.

A mixed ANOVA with the between subject factor group (CC, VM) and the repeated measures factor viewpoint (straight, oblique, profile viewpoints) was run to test for the effect of viewpoint on action identification. There was neither a main effect of group (*F*_(1,22)_ = 1.577, *p* = 0.22; pooled means: CC = 0.85, SD = 0.08; VM = 0.89, SD = 0.08) nor a main effect of viewpoint (*F*_(2,44)_ = 1.18, *p* = 0.317, pooled means: straight ahead = 0.85, SD = 0.11; oblique = 0.89, SD = 0.11; profile = 0.87,SD = 0.09). Further, the interaction of group and viewpoint was non-significant (*F*_(2,44)_ = 0.942, *p* = 0.397; Fig. 4).

### Effect of duration of visual deprivation on task performance

**Figure 5.**
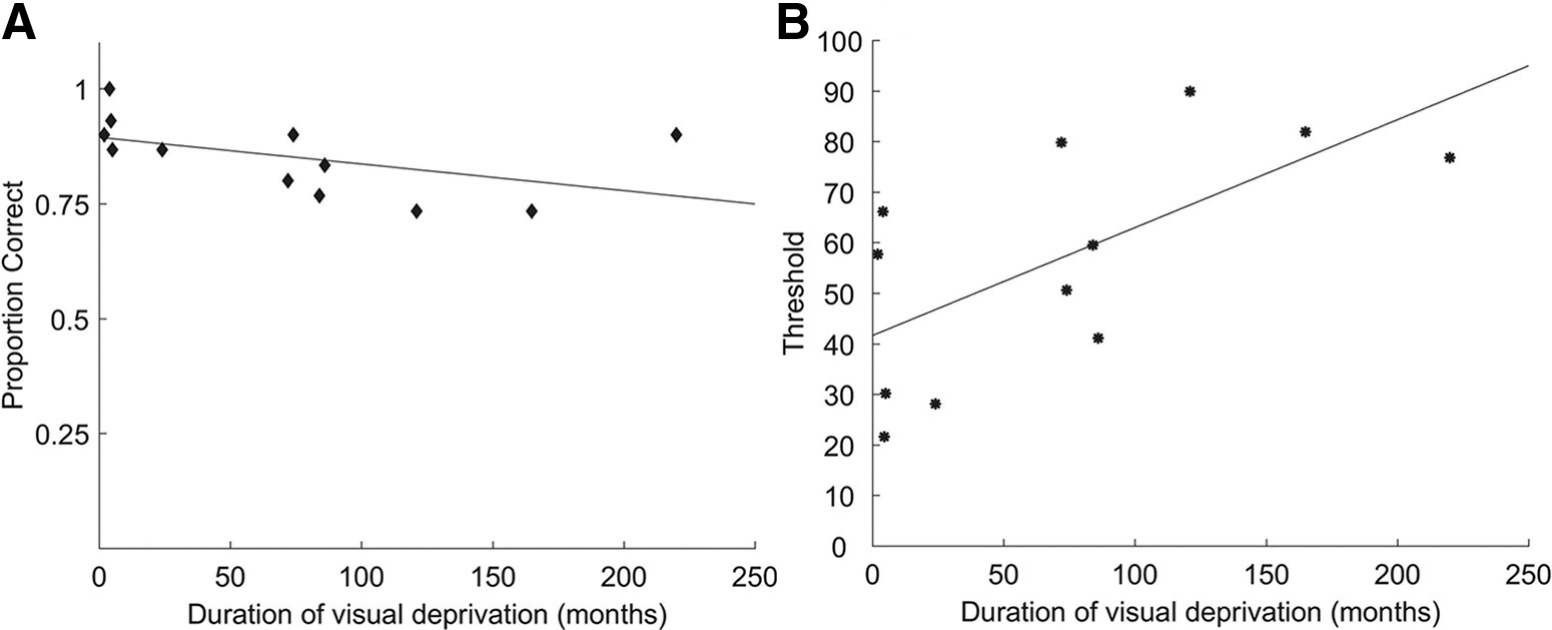
Scatter plot showing the performance of CC group in the (***A***) biological action identification task and (***B***) coherence motion detection task as a function of the duration of visual deprivation. Data points indicate single subjects. The data were fitted with a linear regression line (for definitions, see Fig. 1).

A partial correlation between the duration of visual deprivation and the performance in biological action identification task after controlling for the duration of sight recovery following cataract surgery was not significant (Pearson’s *r*_(10)_ = −0.43; *p* = 0.18; Fig. 5*A*). In contrast, the corresponding partial correlation for the CM task was significant (Pearson’s *r*_(10)_ = 0.63, *p* = 0.03; Fig. 5*B*).

### Effect of VA on CM detection thresholds

**Figure 6.**
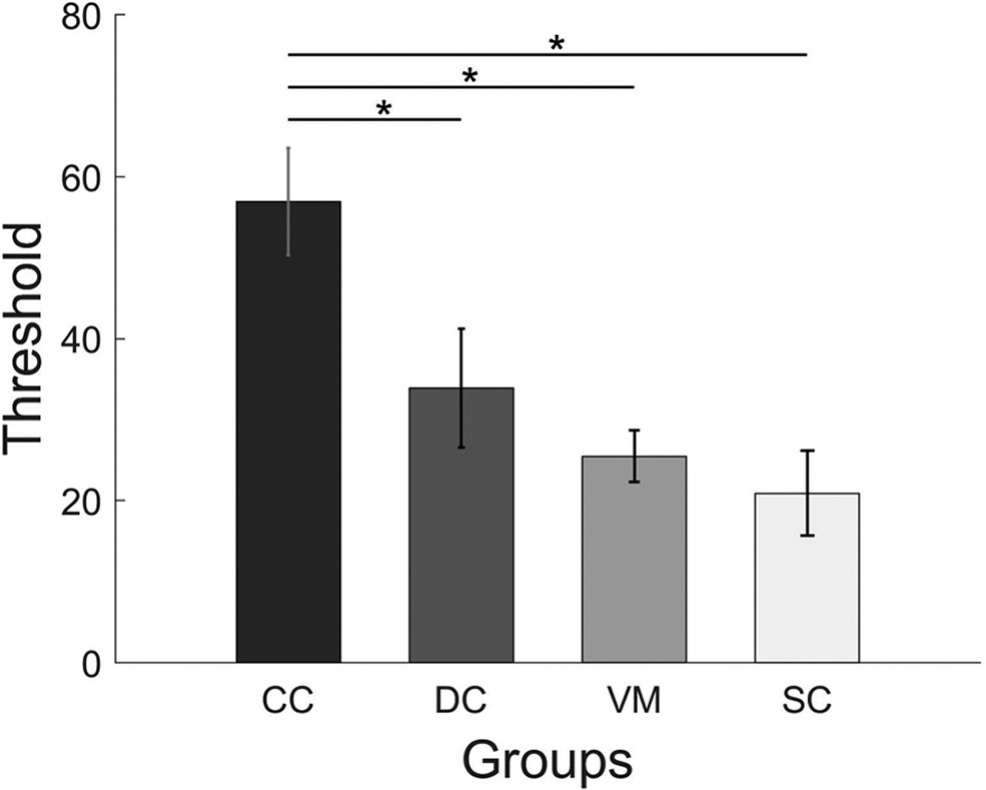
CM thresholds. Bars display CM thresholds in each group; CC, DC, VM, SC (for definitions, see Fig. 1). The *y*-axis specifies the percentage of dots moving coherently to detect the CM with an accuracy of 82%. Shown are mean scores, and the error bars indicate SEM. Asterisks indicate significant group differences.

Detection thresholds for CM were compared between CC, DC, VM groups using a one-way ANOVA (Fig. 4). The group difference was found to be significant (*F*_(2,19.1)_ = 8.87; *p* = 0.001). *Post hoc* group comparisons using Bonferroni corrected *t* tests showed that the CM threshold for the CC group (mean threshold = 56.9, SD = 22.9) was significantly higher than both for the DC (mean threshold = 33.9, SD = 25.4; *p* = 0.03) and the VM group (mean threshold = 25.5, SD = 11.1; *p* = 0.002), while the DC and the VM group did not differ (*p* = 0.98). In a separate analysis, the one-way ANOVA comparing the CC, DC, and SC groups revealed a significant group effect (*F*_(2,33)_ = 7.95; *p* = 0.001). Bonferroni corrected group comparisons found significantly higher CM thresholds for the CC group than for the SC group (mean threshold = 20.9, SD = 18.2; *p* = 0.001). Again, the DC group did not significantly differ from the SC group (*p *=* *0.49). Blurring the visual stimuli did not significantly reduce CM thresholds in the normally SC group (*t*_(11)_ = 0.98, *p* = 0.34) as tested using a paired *t* test comparing the VM and SC groups (Fig. 6).

## Discussion

BM detection thresholds in sight recovery individuals with a history of congenital cataract have been found to be indistinguishable compared with both typical SCs and individuals with a history of DC (Hadad et al., 2012; Bottari et al., 2015). In the present study, we tested a group of individuals with CC (CC group) after cataract removal surgery (a few of whom had had long lasting visual deprivation of up to ∼18 years) in a more complex BM task, that is, the identification of human actions. Three control groups were employed: to test for unspecific effects of cataract surgery and for the importance of visual pattern input at birth, we ran a group of DC reversal individuals (DC group); to test for the effects of persisting VA loss, we included normally SCs both with (VM group) and without (SC group) a blurring of their sight. Additionally, a CM task was performed by the same individuals. Despite some CC individuals still suffering severe acuity loss after cataract removal surgery they were able to recognize human actions with an impressive precision (mean accuracy of 85%). The VM group’s results demonstrated that the slightly but statistically significant lower performance of the CC compared with the SC group could be accounted for by overall lower VAs at the time of testing. This finding for action identification was in stark contrast to the results in the CM task: CC individuals performed worse than all three control groups. This pattern of results in the same individuals provides strong evidence for an impressive resilience of complex BM processing, such as recognizing human actions to aberrant visual experience after birth.

Detecting BM has been shown to be present in human infants (Bertenthal et al., 1987; Simion et al., 2008) and other animals such as chicks (Vallortigara et al., 2005). BM processing does not seem to rely on typical temporal motion processing areas, since lesions in these areas do not affect participants’ ability to perceive BM (Vaina et al., 1990), and their ability to identify actions performed by human point light displays (McLeod, 1996). Here we tested specific actions, almost exclusive of human beings such as bipedal climbing stairs and kicking. Neurons responding to BM and actions have been observed in a number of brain areas such as F5 of the premotor cortex in macaque monkeys (Giese and Poggio, 2003). In macaques, neurons of the superior temporal sulcus (STS) have been shown to respond to full body movement (Oram and Perrett, 1994), and these neurons have been found to be multisensory (Bruce et al., 1981). The action-related (Grossman et al., 2004) and multisensory nature (Bidet-Caulet et al., 2005; Nath and Beauchamp, 2011) of the STS have been demonstrated in humans too. Hence, it could be speculated that during development, these neurons acquire their functional tuning by means of auditory and proprioceptive cues of self-motion. After sight restoration, CC individuals might associate the cues of self-motion from the multisensory neurons with the newly available visual cues, which in turn allows them to learn to recognize visual actions. This idea is compatible with brain imaging studies in congenitally blind humans which observed an activation of premotor and parieto-temporal areas to sounds produced by human actions; the activation overlapped with that found in sighted people for both auditory and visual actions (Ricciardi et al., 2009). Alternatively, it could be argued that the recognition of visual actions is an innate ability of humans possibly because of its high relevance for survival. This idea is expressed in the concept of perceptual life detectors (Troje and Westhoff, 2006). The finding that newborns as young as 2 d preferentially look at biological movement (Simion et al., 2008) is compatible with this idea. However, detecting BM does not mean that infants were able to recognize actions. In particular, actions which involve some artefacts, such as kicking or catching a ball seem to be unlikely innate. A third alternative is that the ability to identify human actions was predominantly visually learned after sight restoration. We did not find a significant correlation between duration of deprivation and performance in the biological action identification task after taking the duration since cataract removal into account (partial correlation Pearson’s *r*_(10)_ =−0.43; *p* = 0.18). Although we hesitate to interpret a null finding in a relatively small sample size, this finding is at least compatible with the idea that the exact time of vision restoration does not matter for learning to recognize human actions as would be predicted by the existence of a sensitive period. Humans might thus be predisposed to learn biological actions. It might be argued that remaining pre-surgery vision had allowed CC individuals to acquire knowledge of typical BM patterns for human actions. However, most CC participants did not have useful pre-surgery vision and the one with useful vision had absorbed lenses (suggesting that the lenses had been dense at birth). Moreover, this participant (CC12) and a second participant with more than light sensation before surgery (CC4) belonged to the worst performing CC individuals (76% and 73% correct, respectively), likely because of their relatively low postsurgery VA (see Table 1).

Another astonishing finding of the present study was the ability of CC individuals to recognize human actions independent of the viewpoint similarly as found for the control groups. This is a remarkable finding, since it has been reported that CC individuals have impairments in recognizing faces from different viewpoints (Putzar et al., 2010; de Heering and Maurer, 2014). In fact, the latter causes a severe impairment in CC individuals in recognizing people by their face in everyday situations. Neuroscience studies have provided evidence that neural systems typically specialized for face processing, including the extraction of face invariant features, lack the functional specificity in CC individuals (Röder et al., 2013; Grady et al., 2014). Since face processing improves with functional differentiation of the fusiform gyrus, a core area of the face processing system (Cantlon et al., 2011), it was speculated that a non-specialized neural system allows for simple functions like face detection and matching but not for the extraction of face invariant features which would allow for the face identity recognition in different contexts. Interestingly, Bottari et al. (2015) demonstrated in the same CC individuals who did not show a face specific neurophysiological response, a selective event-related potential response for intact versus scrambled BM, suggesting that the neural systems of BM processing acquired a functional specialization despite early visual deprivation. These lines of evidence suggest that the neural system for face and BM processing can be dissociated. In fact, a recent study in patients with lesions in the ventral temporal cortex including the fusiform gyrus found that they had intact BM perception (Gilaie-Dotan et al., 2015) including action identification despite their severe impairments in face processing. Further, controlling for VA, the present study suggests that low vision somewhat attenuates action identification but that action identification is still high even under low VA conditions.

In contrast to BM processing, CM processing was impaired in the CC group. In accordance with previous reports (Burton et al., 2015), we found that CM processing threshold did not decrease by blurring the stimuli in the VM group. Thus, the deficit observed in CC individuals cannot be explained by trivial VA differences. Moreover, CM thresholds varied with the age at surgery in the CC individuals: the longer their visual deprivation had lasted, the worse their performance in the CM task. In the context of indistinguishable CM thresholds in the SC and VM group, the present results suggest that the CM processing deficits of the CC individuals must be because of changes in the neural circuits associated with CM processing. In fact, a previous EEG study reported that α oscillations which have been associated with global motion processing (Händel et al., 2007) were greatly reduced in CC individuals (Bottari et al., 2016). It could be argued that the selective deficit in CM perception of CC individuals was predominantly because of the presence of nystagmus in this group. Such deficits for nystagmus patients (with other etiologies than congenital cataract) have mostly been found for slower motion velocities than used in the present study (Shallo-Hoffmann et al., 1998; Neveu et al., 2009). One study (but see Shallo-Hoffmann et al., 1998) observed deficits particular for vertical motion velocity discrimination, that is, motion perpendicular to the direction of the nystagmus (Neveu et al., 2009). However, vertical motion processing deficits would predict a particular impairment of CC individuals for BM tasks as used in the present and in a previous study (Bottari et al., 2016) which unlike the CM task comprised vertical motion components. However, this is not what was found in the present study. Further, despite the presence of nystagmus in all participants CM thresholds considerably varied in the CC group. Therefore, while it is not possible to entirely exclude some effect of nystagmus on CM processing, it seems rather unlikely that the presence of nystagmus could explain the current pattern of results. Finally, given the relatively low number of dots used in the CM detection task, it could be claimed that the CC group did not use motion cues as effectively as the SC group. This is unlikely because the VM group used such cues similarly efficiently as the SC group. Additionally, to use a dot size compatible with the participants’ VA, the number of dots in the CM display was much lower than in most previous studies investigating the motion coherence thresholds. This may have limited the lowest thresholds that could have been measured in the SC and VM groups. However, the higher thresholds of the CC group would not have been subject to this limitation, and so the difference of coherence thresholds between groups as observed in the present study, if affected, might be an underestimate.

It might be argued that the performance in the biological action identification task was at ceiling and thus the task was much easier than the CM task resulting in the simple dissociation observed in the present study. Of course, it is not possible to compare the outcome of the two tasks on the same scale. However, we consider this account for the pattern of results in the present study as unlikely: First, the biological action identification task, unlike the CM task worsened by blurring. Moreover, some items were more difficult than others (e.g., “catching” was the most difficult) demonstrating that performance was not at ceiling. Interestingly, the relative ease or difficulty of action identification for each item was consistent across groups, suggesting the involvement of similar processing mechanisms. Second, the high performance in the biological action identification task of CC individuals was in accordance with indistinguishable thresholds to detect BM (Hadad et al., 2012; Bottari et al., 2015). Third, global motion processing has been shown to be impaired in a number of developmental disorders such as fragile X, preterm infants, dyslexia, amblyopia and hemiplegia (for review, see Braddick et al., 2017), and these findings have led to the suggestion that dorsal stream functions are highly vulnerable during development (Braddick et al., 2003). The present results of the CC group are in line with this idea. Vulnerability to visual deprivation is the downside of high experience dependence and plasticity. Therefore, we would expect that individuals who particularly rely on visual motion processing from early on, such as congenitally deaf individuals, make use of this enhanced plasticity to acquire an extraordinarily high ability in visual motion processing. In fact, lower thresholds for global motion detection (Neville and Lawson, 1987) and enhanced neural processing (Hauthal et al., 2013; Retter et al., 2019) have been observed in deaf humans (Neville et al., 1983). As suggested by Stevens and Neville (2009), sensitive periods are a double edged sword: the lack of experience results in permanently impaired functioning and a particularly challenging stimulation results in enhanced functioning.

In sum, we demonstrate an impressive sparing of a complex visual function, that is, the identification of human actions in a group of sight recovery individuals who had suffered a congenital loss of pattern vision for up to 18 years. By controlling for VA as well as for unspecific effects of cataract surgery and the onset of visual deprivation, we furthermore provide in the same individuals strong additional evidence for an experience dependent development of CM processing during a sensitive period in early ontogeny.

## Synthesis

Reviewing Editor: Tatiana Pasternak, University of Rochester Decisions are customarily a result of the Reviewing Editor and the peer reviewers coming together and discussing their recommendations until a consensus is reached. When revisions are invited, a fact-based synthesis statement explaining their decision and outlining what is needed to prepare a revision will be listed below. The following reviewer(s) agreed to reveal their identity: Oliver Braddick, Daphne Maurer.

Both reviewers appreciated the importance of the problem examined in the paper. However, they raised a number of serious issues that must be addressed for the manuscript to be considered for publication in eNeuro. These are listed below.

1. Please address the problem of comparing the performance on the two tasks that were not equated for difficulty. It would have been more appropriate to use coherence levels at which the subjects perform at comparable % correct in both tasks, as Reviewer 1 suggests.

2. Please provide information about the age of cataract onset in the DC group by adding the data to Table 1. Importantly, more detailed information about the CC group should be provided, as suggested by Reviewer 2 , who pointed out the heterogeneity of this group. Also, please explain the criteria for including patients in the CC rather the DC group.

3. Please comment on the refractive state of the patients during testing and before surgery and whether nystagmus was still present during behavioral testing

4. Please clarify whether the four groups were geographically and culturally similar

5. It appears from the text that only 15 dots were presented for 1 second. If this is a correct description, it raises the question the question concerning the cues used by the SC group to identify motion direction and whether CC and DC patients perform poorly because of the inability to utilize such cues. This question should be discussed.

6. Please provide proper references. Both reviewers stress incompleteness of references and numerous mistakes and typos.

7. Please make sure to address all the minor points raised by each reviewer. REVIEWER 1

ADVANCES IN THE FIELD

Characterises more fully the visual abilities and impairment of individuals who had no visual experience during early life - a key area for understanding the plasticity of the human visual system

COMMENTS TO THE AUTHORS

This paper discusses an important issue in visual development and plasticity - what is the relative vulnerability to deprivation of two different kinds of motion-based visual performance: (a) the discrimination of actions in moving point-light displays and (b) coherence thresholds for discriminating motion direction. The comparison of performance by patients following repair of congenital cataracts, later acquired cataracts, and healthy controls with acuity reduced to match the patients, provides evidence that task (b) is much more impaired by early deprivation than (a). This difference can be linked to the different cortical pathways involved in the two tasks.

The work is well designed and executed and deserves publication. However, there are a number of issues that the authors need to address.

1. The measures for the two tasks are rather different: % correct on a fixed set of stimuli for (a) vs threshold determined by an adaptive staircase for (b). This raises a question of how validly the two measures can be compared, and in what sense the difficulty of the two tasks is commensurate. Performance on (a) is not quite at ceiling, but the approximately 80% correct could be achieved by perfect performance by all groups on identifying most of the 30 stimuli (10 actions) and complete failure on 1 or 2 actions. This would lead to the observed result in a test which was not really sensitive to possible group differences. It would be helpful to have an indication of how far different levels of performance on the 10 different actions might underlie the pattern of results.

2. An alternative approach to the question raised above would be to produce a more comparable performance measure on the motion coherence task. Suppose we take a coherence level on which the VM group achieve a certain level, say 80% correct. Would it be possible to estimate the performance of the cataract groups with this same coherence level? I appreciate that the adaptive staircase will not yield this directly, but the QUEST method should generate an estimate of the slope of the psychometric function from which this coherence level could be estimated, and this would give a plot for comparing coherence sensitivity more commensurate with Figure 2.

3. For the argument about sensitive periods of development, the age of cataract onset in the DC group is critical. This is not stated even in the summary description. Ideally a table of DC individuals should be included including this data and the other information provided in Table 1 for the CC group.

4. I presume that both the CC and DC patients wore their best refractive correction for testing? This is not stated, and should be. It would also be important to have some estimate of the pre-surgical acuity of these patients, if available.

5. Table 1 lists 'nystagmus' for all the CC patients. State explicitly whether this was simply a diagnostic feature at the time the cataract was present, or whether it persisted at the time of testing (and whether it was present in any of the DC group)

6. While I appreciate the policy that the origin of the participant groups is not disclosed, it is important to be assured of the comparability of these groups. Did they all, including the SC group, come from a similar geographical and cultural background?

7. Two issues about the stimulus presentation.

(a) it is stated that the motion coherence stimuli contained 15 dots. This is a very small number compared to the hundreds of dots in typical tests of this kind, and it's not clear how it could be used when testing coherence levels below 20% where the 'signal' would consist of only 1-3 dots.

(b) by my calculation for the on-screen acuity test, the pixel size was about 0.25 mm or 3-4 minarc at 240 cm viewing distance. It does not seem possible for this to be used in measuring acuity for the SC group whose resolution is better than 1 minarc, and would not accurately characterise acuities of 0.5 logMAR which would correspond to the limit of the screen

8. The preparation of the reference list is poor - many lack volume and page numbers, and it is not made clear that at least one (Maurer and Lewis, 2018) is a book chapter (I wasted some time trying to find the journal!)

Some more minor specific points for correction:

The term 'partially severe visual impairment' is used several times - it's not clear what this means.

For the work of Lewis and Ellemberg on motion coherence for patients with cataracts repaired in infancy, only the review paper by Hadad is cited. It would be better to include the original 2002 paper (and perhaps their second paper which allows a direct compassion with (less affected) form coherence.

Line 65 'sighed' should be 'sighted'

The work of Burton et al (2015, Journal of Vision 15(15):12, 1-14) should be cited as supporting evidence that reduced acuity has a relatively small impact on motion coherence thresholds. Work form the same group on patients with a congenital loss of cone vision(Burton et al, 2016, Invest Ophthal Vis Sci 57:2251-2259) provides an interesting complement to the present results, showing impairment of motion coherence but no biological motion in the scotopic range, and should also be cited.

Lines 288-298 -the argument is unclear. Some of the neurophysiological studies cited refer to neural responses to visual presented body motion (eg Oram and Perrett) while others may relate to 'mirror neurons' which show responses both to viewed motion and self-motion - these are not distinguished in the text. It is not explained how somatosensory responses to self-motion, acquired during blindness, become associated to the visual stimuli of other people's motion once vision is restored.

REVIEWER 2

ADVANCES IN THE FIELD

Small--very nicely designed study confirming a pattern previously shown in the literature and probing the pattern more deeply.

COMMENTS TO THE AUTHORS

This is a nicely designed paper investigating sensitivity to global motion versus biological motion in patients treated for a period of visual deprivation from cataracts. Previous studies have shown large deficits in global motion if the deprivation was congenital (but not if it started postnatally, even during infancy) and a surprising sparing of sensitivity to biological motion. This study probed these patterns more deeply by retesting sensitivity to global motion and requiring that the specific action portrayed by biological motion be recognized. The patterns of deficit for global motion were replicated but all groups were normal at this more sensitive probe of sensitivity to biological motion. The methodology and comparison groups are sophisticated (e.g., controls are tested with normal vision and with their visual acuity degraded to match that of a specific patient to whom they were age-matched).

The paper might make a nice, small addition to the literature if the problems described below can be overcome. The major problems are as follows:

1. The congenital group is very heterogeneous. Some patients had 2-5 months of deprivation 9-20 years ago; others had 10+ years of deprivation 1-2 years ago. If the deprivation in each case had been complete, the outcomes would be expected to be different-the especially long deprivations would be expected to have terrible acuity, likely just CFs, but that is not what is shown in Table 1. More likely is that some of the cataracts, especially those not removed until the child was out of infancy were not dense and central at birth but that they developed postnatally from initially partial cataracts. When a child presents with cataracts of no known origin, ophthalmologists often label them as congenital no matter what the child's age. And if the study was conducted in the developing world, medical records are often quite incomplete. The authors need to address this concern and indicate why one can be confident about the numbers in Table 1. They also need to analyze the data within each group to show if there is any effect of duration of deprivation or of acuity at the time of test.

A related concern is that at line 89, the authors state that patients in the CC group had "total or near total" cataract prior to surgery. They need to be explicit about the criterion for going into the CC group (total or near total) versus the DC group (partial). This is critical to the interpretation of the data as to the type of visual experience required early in infancy. They also state at line 90 that there had to "invisibility of fundus due to media opacity caused by the cataracts". Was that recorded for every case? At line 91, they state that there had to be "a family history of congenital cataract". Was that true in every case? Did every patient meet all of these criteria or was it sufficient to meet some of them? Which criteria had to be missed in order to be classified DC instead?

2. More attention and care should be paid to details when preparing the manuscript. For example, a good 1/3 of the references are incomplete. Particularly alarming is that the manuscript refers repeatedly to "partially, long lasting congenital cataract (up to 14 years, CC group)". But the patients with partial cataracts, even if present from birth, were placed in the developmental cataract (DC) group. And if the authors meant "particularly long", this is an inaccurate description because Table 1 indicates that 4 patients had deprivation of less than 1 year, 3 had deprivation of 1-2 years, and only 5 had especially long deprivation. There are also many typos.

3. The authors need to give more information about the developmental group, including a table like Table 1. Otherwise, it is not clear how they can draw the conclusion in the last sentence about a sensitive period for global motion.

4. The authors need to consider how the nystagmus present in all of the congenital patients may affected their sensitivity to horizontal global motion and whether it can account for the differences among groups, independent of the differences in early experience.

5. The manuscript is repetitive and much too long for the size of its contribution to the literature.

Minor points:

1. In the Significance statement, it would be better to say that biological motion "emerges" or "is evident" early in development because it continues to improve over 12+ years.

2. The control subjects are not exactly matched on sex (line 108), although it is close enough not to matter.

3. At line 146, the authors indicate that participants (presumably the congenital cataract patients) were allowed to move closer to the screen if the stimuli were not visible at the testing distance of 60 cm. Were their vision-matched controls also allowed to move closer? Was this true for both tasks? How often did this happen and did it have any apparent impact on the results?

4. The studies mentioned at lines 308-309 were not with infants.

---------

## Author Response

Synthesis Statement for Author (Required):

Both reviewers appreciated the importance of the problem examined in the paper. However, they raised a number of serious issues that must be addressed for the manuscript to be considered for publication in eNeuro. These are listed below.

1. Please address the problem of comparing the performance on the two tasks that were not equated for difficulty. It would have been more appropriate to use coherence levels at which the subjects perform at comparable % correct in both tasks, as Reviewer 1 suggests.

We used the biomotion test battery (BMT), which was available online. The BMT had been used in previous research (Bottari et al., 2018, 2015) and had been validated (Saunders and Troje, 2011). For the coherent motion test the threshold was set to 82% correct performance as defined by a QUEST staircase method. Staircase procedures are efficient to derive the threshold with a minimum number of trials, but since most measurements are taken close to threshold, they do not provide a reliable measure of the slope of the psychometric function. Our participants were primarily recruited during a regular follow-up visit to the clinic and thus, the time we were able to spend collecting data was limited.

Unfortunately, we do only have access to the final threshold measure which is the output of this test. However, the final threshold comes close to what is suggested by this comment: 82% correct performance is achieved on average with a coherence of 20.9% in the SC group, of 25.5% in the VM group, of 33.9% in the DC group and of 56.9% in the CC group. In the revised manuscript this is more explicitly pointed out.

We stress in the revised manuscript the astonishing high ability of CC individuals to recognize human actions and that the slightly lower performance in the CC groups could be accounted for by their lower visual acuity (that is, no significant difference between the CC and the VM group). By contrast, for the coherent motion task the lower performance of the CC participants cannot be explained by visual acuity. (eg - lines 350-353)

2. Please provide information about the age of cataract onset in the DC group by adding the data to Table 1. Importantly, more detailed information about the CC group should be provided, as suggested by Reviewer 2 , who pointed out the heterogeneity of this group. Also, please explain the criteria for including patients in the CC rather the DC group.

The DC group could offer a unique opportunity to narrow down sensitive periods for coherent motion processing. However, developmental cataracts typically emerge slowly which makes it impossible to define an "age at onset". While the date of surgery is available for all the DC participants, the exact date of the onset of cataract is not possible to calculate; the only information available are reports of the parents, which are not sufficiently reliable and do not inform about the degree of the impairment and its time course.

We selected the CC participants from a large group of patients which were classified as "congenital cataracts". As described in the revised manuscript (page 5) we applied a number of criteria to accumulate evidence for the presence of dense bilateral and total cataracts at birth.

Our criteria for classifying participants into the CC group included the presence of nystagmus, and a lenticular opacity dense enough (total or near total cataract) to prevent the view of the fundus. All participants for whom family history was available had a positive family history of congenital cataract. Cases for which clinical signs were not clear and classification became ambiguous were not included. Moreover, the presence of esotropia, helped us classifying the CC individuals, since estoropia typically emerges if both eyes are equally affected after birth (Havertape et al., 2001).

We would like to stress, that we consider the "heterogeneity" of our CC group with regard to age at surgery (but NOT with regard the etiology) as an advantage of our study underlining the crucial role of the visual experience during the first weeks of life for coherent motion perception but the resilience of human action perception to even long-lasting pattern vision loss. As for CC participants, the DC participants were selected from a large group of patients which were classified as "developmental cataracts". These patients typically did not experience nystagmus suggesting the presence of early visual experience. Three patients who had non-dense congenital cataract prior to surgery were grouped as DC individuals too. This has been made clearer in the methods section - "participants". (lines 87-98)

3. Please comment on the refractive state of the patients during testing and before surgery and whether nystagmus was still present during behavioral testing

Nystagmus was present in all CC participants during testing. This information from Table 1 has been added to the main text of the manuscript (lines 90,91). All participants wore their habitual refractive correction during testing. This information has been added in the methods section (lines 193-194).

4. Please clarify whether the four groups were geographically and culturally similar.

Yes, they were geographically and culturally similar. We think that this will become clear in the final version once the location of the place of testing will be revealed (lines 85, 106, 114).

5. It appears from the text that only 15 dots were presented for 1 second. If this is a correct description, it raises the question the question concerning the cues used by the SC group to identify motion direction and whether CC and DC patients perform poorly because of the inability to utilize such cues. This question should be discussed.

Since the majority of participants were expected to have reduced vision, we had to enlarge the size of the dots (each dot was 15 pixels in diameter) in order to ensure that the dots could be well seen. As a consequence we had to reduce the number of dots. As seen in the results, all participants of all groups were able to make use of the moving dots but crucially at different coherence levels. Bottari et al (2018) used the same stimuli (dot number, size and stimulus duration). This has been made more explicit in the revised manuscript (line 165). Their ERP study demonstrated that for both DC and SC groups ERPs (the N170) differed for low and high coherence levels, suggesting a valid manipulation of coherence levels.

Further, the SC and DC groups differed from the CC group in their absence of nystagmus, good visual acuity, and presence of early visual input. Visual acuity was accounted for by using a visual matched SC group (using blurring glasses). Deficits for nystagmus patients (with other aetiologies than congenital cataract) have mostly been found for slower motion velocities than used in the present study (Neveu et al., 2009; Shallo-Hoffmann et al., 1998). One study (but see Shallo-Hoffmann et al, 1998) observed deficits particular for vertical motion velocity discrimination, that is, motion perpendicular to the direction of the nystagmus (Neveu et al., 2009). However, vertical motion processing deficits would predict a particular impairment of CC individuals for biological motion tasks as used in the present and in a previous study (Bottari et al., 2016) and not for coherent motion task which only comprised horizontal motion components. This has been discussed in the revised manuscript lines 357 -366.

Thus, we concluded that the lack of early visual input is the most likely cause for the results in our CC population. This information has been added in the discussion (lines 366-372)

6. Please provide proper references. Both reviewers stress incompleteness of references and numerous mistakes and typos.

The list of references of the revised manuscript has been compiled with a different software and was thoroughly proof read.

7. Please make sure to address all the minor points raised by each reviewer.

All minor comments of the reviews have been addressed in the revised manuscript.

---------

REVIEWER 1

ADVANCES IN THE FIELD

Characterises more fully the visual abilities and impairment of individuals who had no visual experience during early life - a key area for understanding the plasticity of the human visual system

COMMENTS TO THE AUTHORS

This paper discusses an important issue in visual development and plasticity - what is the relative vulnerability to deprivation of two different kinds of motion-based visual performance: (a) the discrimination of actions in moving point-light displays and (b) coherence thresholds for discriminating motion direction. The comparison of performance by patients following repair of congenital cataracts, later acquired cataracts, and healthy controls with acuity reduced to match the patients, provides evidence that task (b) is much more impaired by early deprivation than (a). This difference can be linked to the different cortical pathways involved in the two tasks.

The work is well designed and executed and deserves publication. However, there are a number of issues that the authors need to address.

1. The measures for the two tasks are rather different: % correct on a fixed set of stimuli for (a) vs threshold determined by an adaptive staircase for (b). This raises a question of how validly the two measures can be compared, and in what sense the difficulty of the two tasks is commensurate. Performance on (a) is not quite at ceiling, but the approximately 80% correct could be achieved by perfect performance by all groups on identifying most of the 30 stimuli (10 actions) and complete failure on 1 or 2 actions. This would lead to the observed result in a test which was not really sensitive to possible group differences. It would be helpful to have an indication of how far different levels of performance on the 10 different actions might underlie the pattern of results.

As seen in Figure A (see below), although responses varied across actions, the CC group did not totally fail in any of the actions.

The worst mean performance of the CC group was observed for "catching" (mean accuracy=67%). However, both the DC and the VM group preformed similarly bad for the "catching" action while none of the groups totally failed. This information has been added to the result section. Given, that we did not have any prior hypothesis about single action items and possible interactions with the group factor, we refrained from running any post hoc statistics. Nevertheless, in order to address the issue of possible ceiling effects, Figure A has been included in the revised manuscript as Figure 2b.

Figure A. Bar plots displaying the proportion of correct responses for each group: CC (Congenital Cataract), DC (Developmental Cataract), VM (Vision Matched), SC (Sighted Controls) averaged for each of the 10 actions

2. An alternative approach to the question raised above would be to produce a more comparable performance measure on the motion coherence task. Suppose we take a coherence level on which the VM group achieve a certain level, say 80% correct. Would it be possible to estimate the performance of the cataract groups with this same coherence level? I appreciate that the adaptive staircase will not yield this directly, but the QUEST method should generate an estimate of the slope of the psychometric function from which this coherence level could be estimated, and this would give a plot for comparing coherence sensitivity more commensurate with Figure 2.

The main finding of the present study was the astonishing high accuracy in detecting biological actions in CC individuals while the very same participants were impaired in coherent motion processing, even when visual acuity was controlled. We are aware of the fact, that we demonstrated a simple and not a double dissociation. Thus, it could be argued that the result pattern could be due to simply the bioAction task having been too easy to reveal group differences (ceiling effect). We consider this option as highly unlikely: First, the DC and VM groups did not perform at ceiling, nor did the SC group with most of the items (see Figure A). Second, for other biological motion related tasks such as detection tasks, previous studies observed highly preserved performance in the CC group too (see (Bottari et al., 2015) and (Hadad et al., 2012)). Bottari et al (2015) used a similar QUEST procedure implemented in the BioMotion Test battery (BMT) to assess the threshold for detecting biological motion (a walking point light person). In this test the degree of randomly moving dots was systematically manipulated. Thresholds did not differ between CC, DC and SC groups. Here we extended this finding to a more complex biological motion task requiring the identification of human actions.

We used the biomotion test battery (BMT), which was available online. The BMT had been used in previous research (Bottari et al., 2018, 2015) and had been validated (Saunders and Troje, 2011). For the coherent motion test the threshold was set to 82% correct performance as defined by a QUEST staircase method. Staircase procedures are efficient to derive the threshold with a minimum number of trials, but since most measurements are taken close to threshold, they do not provide a reliable measure of the slope of the psychometric function. Our participants were primarily recruited during a regular follow-up visit to the clinic and thus, the time we were able to spend collecting data was limited.

Unfortunately, we do only have access to the final threshold measure which is the output of this test. However, the final threshold comes close to what is suggested by this comment: 82 % correct performance is achieved on average with a coherence of 20.9% in the SC group, of 25.5% in the VM group, of 33.9% in the DC group and of 56.9% in the CC group. In the revised manuscript this is more explicitly pointed out.

We stress in the revised manuscript the astonishing high ability of CC individuals to recognize human actions and that the slightly lower performance in the CC groups could be accounted for by their lower visual acuity (that is, no significant difference between the CC and the VM group). By contrast, for the coherent motion task the lower performance of the participants cannot be explained by visual acuity. (eg - lines 350-353)

3. For the argument about sensitive periods of development, the age of cataract onset in the DC group is critical. This is not stated even in the summary description. Ideally a table of DC individuals should be included including this data and the other information provided in Table 1 for the CC group.

The DC group could offer a unique opportunity to narrow down sensitive periods for coherent motion processing. However, developmental cataracts typically emerge slowly which makes it impossible to define a "age at onset". While the date of surgery is available for all the DC participants, the exact date of the onset of developmental cataract is not possible to calculate; the only information available are reports of the parents, which are not sufficiently reliable and do not inform about the degree of the impairment and its time course.

4. I presume that both the CC and DC patients wore their best refractive correction for testing? This is not stated, and should be. It would also be important to have some estimate of the pre-surgical acuity of these patients, if available.

All patients wore their best refractory corrections. This information has been added in the methods section. Lines 193-194.

We added the presurgery acuity to the majority of congenital cataract-reversal individuals in Table 1 of the revised manuscript. For 3 patients this information was not available because these patients had received surgery at another hospital and were diagnosed based on the criteria listed in methods section - participants. For 2 other patients, the information is in the printed medical records. Due to the lockdown in the country where the study was conducted, this department has not been working in the past weeks and it is not known when the staff will return. However, these individuals meet other criteria listed in methods section.

5. Table 1 lists ‘nystagmus’ for all the CC patients. State explicitly whether this was simply a diagnostic feature at the time the cataract was present, or whether it persisted at the time of testing (and whether it was present in any of the DC group)

Congenital visual deprivation which is not removed prior to the age of 10-13 weeks (Gelbart et al., 1982; Rogers et al., 1981) , results in a persisting nystagmus. In fact, the presence of nystagmus after cataract reversal surgery is often used as one diagnostic criterion for congenital cataract. The criteria for DC (lines 107-111) and CC (lines 87-98) classification have been made more explicit in the methods section.

6. While I appreciate the policy that the origin of the participant groups is not disclosed, it is important to be assured of the comparability of these groups. Did they all, including the SC group, come from a similar geographical and cultural background?

Yes, all participants came from similar geographical and cultural backgrounds. This will become more apparent once the location of the study will be revealed in the final version of the manuscript.

7. Two issues about the stimulus presentation.

(a) it is stated that the motion coherence stimuli contained 15 dots. This is a very small number compared to the hundreds of dots in typical tests of this kind, and it’s not clear how it could be used when testing coherence levels below 20% where the ‘signal’ would consist of only 1-3 dots.

Since we expected that most of the participants would have reduced vision, we had to enlarge the size of the dots (each dot was 15 pixels in diameter) in order to ensure that the dots would be easily visible to all participants. The threshold for coherence motion detection in CC individuals was at ∼57% (range 22% to 90%). None of our CC participants had thresholds less than 13.33% where the signal would have been 2 dots or less. However, for the SC (mean ∼21%; range: 5% to 62%) and VM group (mean ∼26%; range: 7% to 49%) the coherent motion threshold might have been overestimated. Thus, the observed group differences are if at all an underestimation of the real impairment of the CC group.

(b) by my calculation for the on-screen acuity test, the pixel size was about 0.25 mm or 3-4 minarc at 240 cm viewing distance. It does not seem possible for this to be used in measuring acuity for the SC group whose resolution is better than 1 minarc, and would not accurately characterise acuities of 0.5 logMAR which would correspond to the limit of the screen

The settings were based on recommendations given by the FrACT software . At 240 cm each pixel subtended 0.39 mins of an arc. This is based on the calculations in the manual (https://michaelbach.de/fract/media/FrACT3_Manual.pdf - especially page 9). With our settings the max decimalVA that could be achieved was 2.26. By standard conversions (https://pdfs.semanticscholar.org/9645/6b86671320880d6e5e7e9e1745d250a35224.pdf) this is negative 0.35 (-0.35) logMAR (eg - https://michaelbach.de/sci/acuity.html) which is much higher than the best visual acuity of 0.00 logMAR. The minimum acuity in our SC was 0.00 logMAR and the maximum VA was -0.3. This is within the range of visual acuities that the display is calibrated for.

8. The preparation of the reference list is poor - many lack volume and page numbers, and it is not made clear that at least one (Maurer & Lewis, 2018) is a book chapter (I wasted some time trying to find the journal!)

The list of references of the revised manuscript has been compiled with a different software and was thoroughly proof read.

Some more minor specific points for correction:

The term ’partially severe visual impairment’ is used several times - it’s not clear what this means.

We meant to say that some of our participants had still severe visual impairments after surgery but were nevertheless able to perform the bioAction task well. In the revised manuscript the term ‘partially severe visual impairment’ has been replaced by ‘some CC individuals still suffering severe acuity loss after cataract removal surgery’.

For the work of Lewis and Ellemberg on motion coherence for patients with cataracts repaired in infancy, only the review paper by Hadad is cited. It would be better to include the original 2002 paper (and perhaps their second paper which allows a direct compassion with (less affected) form coherence.

Ellemberg et al’s 2002 paper has been added

Line 65 ‘sighed’ should be ‘sighted’

corrected

The work of Burton et al (2015, Journal of Vision 15(15):12, 1-14) should be cited as supporting evidence that reduced acuity has a relatively small impact on motion coherence thresholds. Work form the same group on patients with a congenital loss of cone vision(Burton et al, 2016, Invest Ophthal Vis Sci 57:2251-2259) provides an interesting complement to the present results, showing impairment of motion coherence but no biological motion in the scotopic range, and should also be cited.

The work of Burton et al. has been included. Eg - lines 351

Lines 288-298 -the argument is unclear. Some of the neurophysiological studies cited refer to neural responses to visual presented body motion (eg Oram & Perrett) while others may relate to ‘mirror neurons’ which show responses both to viewed motion and self-motion - these are not distinguished in the text. It is not explained how somatosensory responses to self-motion, acquired during blindness, become associated to the visual stimuli of other people’s motion once vision is restored.

The argument has been differently phrased. Lines 302-305

REVIEWER 2

ADVANCES IN THE FIELD

Small--very nicely designed study confirming a pattern previously shown in the literature and probing the pattern more deeply.

COMMENTS TO THE AUTHORS

This is a nicely designed paper investigating sensitivity to global motion versus biological motion in patients treated for a period of visual deprivation from cataracts. Previous studies have shown large deficits in global motion if the deprivation was congenital (but not if it started postnatally, even during infancy) and a surprising sparing of sensitivity to biological motion. This study probed these patterns more deeply by retesting sensitivity to global motion and requiring that the specific action portrayed by biological motion be recognized. The patterns of deficit for global motion were replicated but all groups were normal at this more sensitive probe of sensitivity to biological motion. The methodology and comparison groups are sophisticated (e.g., controls are tested with normal vision and with their visual acuity degraded to match that of a specific patient to whom they were age-matched).

The paper might make a nice, small addition to the literature if the problems described below can be overcome. The major problems are as follows:

1. The congenital group is very heterogeneous. Some patients had 2-5 months of deprivation 9-20 years ago; others had 10+ years of deprivation 1-2 years ago. If the deprivation in each case had been complete, the outcomes would be expected to be different-the especially long deprivations would be expected to have terrible acuity, likely just CFs, but that is not what is shown in Table 1. More likely is that some of the cataracts, especially those not removed until the child was out of infancy were not dense and central at birth but that they developed postnatally from initially partial cataracts. When a child presents with cataracts of no known origin, ophthalmologists often label them as congenital no matter what the child’s age. And if the study was conducted in the developing world, medical records are often quite incomplete.

The authors need to address this concern and indicate why one can be confident about the numbers in Table 1. They also need to analyze the data within each group to show if there is any effect of duration of deprivation or of acuity at the time of test.

We controlled for visual acuity by blurring the images for normally sighted individuals, see data of VM group.

Below (Figure B and Figure C) are two graphs plotting the CC individuals’ performance in the bioAction and coherent motion task against age at surgery. Duration of visual deprivation did not correlate with the performance in the bioAction task (partial correlation Pearson’s r (10)= -0.36; p = 0.28) after controlling for the duration of sight recovery after cataract removal; see Figure B There was a trend towards a positive correlation of coherence motion thresholds and the duration of visual deprivation (partial correlation Pearson’s r(10) = 0.54, p = 0.09), after controlling for the duration of visual recovery following cataract removal; Figure C): performance in the coherent motion task tended to decrease with a longer duration of visual deprivation. We refer to the correlation results in the discussion lines 320-325, line 382.

Figure B - Performance in the bioAction task as a function of visual deprivation in the CC group. Circles indicate single subjects. The data have been fitted with a linear regression line

Figure C - Coherence thresholds as a function of the duration of visual deprivation in the CC group. Triangles indicate single subjects. The data have been fitted with a linear regression line.

A related concern is that at line 89, the authors state that patients in the CC group had "total or near total" cataract prior to surgery. They need to be explicit about the criterion for going into the CC group (total or near total) versus the DC group (partial). This is critical to the interpretation of the data as to the type of visual experience required early in infancy. They also state at line 90 that there had to "invisibility of fundus due to media opacity caused by the cataracts". Was that recorded for every case? At line 91, they state that there had to be "a family history of congenital cataract". Was that true in every case? Did every patient meet all of these criteria or was it sufficient to meet some of them? Which criteria had to be missed in order to be classified DC instead?

We selected the CC participants from a large group of patients which were classified as "congenital cataracts". As described in the revised manuscript (lines 87-98), we applied a number of criteria to accumulate evidence for the presence of dense bilateral and total cataracts at birth.

Our criteria for classifying participants into the CC group included the presence of nystagmus, and a lenticular opacity dense enough (total or near total cataract) to prevent the view of the fundus. All participants for whom family history was available had a positive family history of congenital cataract. Cases for which clinical signs were not clear and classification became ambiguous were not included. Moreover, the presence of esotropia, helped us classifying the CC individuals, since estoropia typically emerges if both eyes are equally affected after birth (Havertape et al 2001).

We would like to stress, that we consider the "heterogeneity" of our CC group with regard to age at surgery (but NOT with regard the etiology) as an advantage of our study underlining the crucial role of the visual experience during the first weeks of life for coherent motion perception but the resilience of human action perception to even long-lasting pattern vision loss. As for CC participants, the DC participants were selected from a large group of patients which were classified as "developmental cataracts". These patients typically did not experience nystagmus suggesting the presence of early visual experience. Three patients who had non-dense congenital cataract prior to surgery were grouped as DC individuals too. This has been made clearer in the methods section - "participants" (lines 107-111)

2. More attention and care should be paid to details when preparing the manuscript. For example, a good 1/3 of the references are incomplete. Particularly alarming is that the manuscript refers repeatedly to "partially, long lasting congenital cataract (up to 14 years, CC group)". But the patients with partial cataracts, even if present from birth, were placed in the developmental cataract (DC) group. And if the authors meant "particularly long", this is an inaccurate description because Table 1 indicates that 4 patients had deprivation of less than 1 year, 3 had deprivation of 1-2 years, and only 5 had especially long deprivation. There are also many typos.

The list of references of the revised manuscript has been compiled with a different software and was thoroughly proof read. The mentioned ambiguous parts of the manuscript have been rewritten. (Lines 281,392)

The revised manuscript has been carefully proofread.

For patient selection, see responses above.

3. The authors need to give more information about the developmental group, including a table like Table 1. Otherwise, it is not clear how they can draw the conclusion in the last sentence about a sensitive period for global motion.

The DC group could offer a unique opportunity to narrow down sensitive periods for coherent motion processing. However, developmental cataracts typically emerge slowly, which makes it impossible to define an "age at onset". While the date of surgery is available for all the DC participants, the exact date of the onset of developmental cataract is not possible to calculate; the only information available are reports of the parents, which are not sufficiently reliable and do not inform about the degree of the impairment and its time course.

4. The authors need to consider how the nystagmus present in all of the congenital patients may affected their sensitivity to horizontal global motion and whether it can account for the differences among groups, independent of the differences in early experience.

Deficits for nystagmus patients (with other aetiologies than congenital cataract) have mostly been found for slower motion velocities than used in the present study (Neveu et al., 2009; Shallo-Hoffmann et al., 1998). One study (but see Shallo-Hoffmann et al, 1998) observed deficits particular for vertical motion velocity discrimination, that is, motion perpendicular to the direction of the nystagmus (Neveu et al., 2009) . However, vertical motion processing deficits would predict a particular impairment of CC individuals for biological motion tasks as used in the present and in a previous study (Bottari et al., 2016) and not for coherent motion task which comprised horizontal motion components. This has been discussed in the revised manuscript lines 357 -366.

5. The manuscript is repetitive and much too long for the size of its contribution to the literature.

Parts of the manuscript have been shortened in order to be more concise. The fact, that the revised manuscript is overall slightly longer than the original manuscript is due to the inclusion of additional information.

Minor points:

1. In the Significance statement, it would be better to say that biological motion "emerges" or "is evident" early in development because it continues to improve over 12+ years.

This has been changed accordingly.

2. The control subjects are not exactly matched on sex (line 108), although it is close enough not to matter.

Since after adolescence developmental asynchronies between sexes are unlikely, we made sure that subjects below the age of 18 years were additionally matched for sex. The obtained age match is made clearer in line 117.

3. At line 146, the authors indicate that participants (presumably the congenital cataract patients) were allowed to move closer to the screen if the stimuli were not visible at the testing distance of 60 cm. Were their vision-matched controls also allowed to move closer? Was this true for both tasks? How often did this happen and did it have any apparent impact on the results?

All groups were allowed to move forward for both the tasks. Thus, any possible resulting bias introduced by this procedure would have been the same for all groups and for both tasks. This aspect has been addressed in lines 159-161.

4. The studies mentioned at lines 308-309 were not with infants.

The studies referred to the term "life detectors", this has been made clear in the new version lines 314-316.

-

References:

Bottari D, Kekunnaya R, Hense M, Troje NF, Sourav S, Röder B (2018) Motion processing after sight restoration: No competition between visual recovery and auditory compensation. Neuroimage 167:284-296.

Bottari D, Troje NF, Ley P, Hense M, Kekunnaya R, Röder B (2016) Sight restoration after congenital blindness does not reinstate alpha oscillatory activity in humans. Sci Rep 6:24683.

Bottari D, Troje NF, Ley P, Hense M, Kekunnaya R, Röder B (2015) The neural development of the biological motion processing system does not rely on early visual input. Cortex 71:359-367.

Gelbart SS, Hoyt CS, Jastrebski G, Marg E (1982) Long-term visual results in bilateral congenital cataracts. Am J Ophthalmol 93:615-621.

Hadad B, Maurer D, Lewis TL (2012) Sparing of sensitivity to biological motion but not of global motion after early visual deprivation. Dev Sci 15:474-481.

Havertape SA, Cruz OA, Chu F (2001) Sensory strabismus - Eso or Exo? J Pediatr Ophthalmol Strabismus 38:327-330.

Neveu MM, Jeffery G, Moore AT, Dakin SC (2009) Deficits in local and global motion perception arising from abnormal eye movements. J Vis 9:1-15.

Rogers GL, Tishler CL, Tsou BH, Hertle RW, Fellows RR (1981) Visual acuities in infants with congenital cataracts operated on prior to 6 months of age. Arch Ophthalmol 99:999-1003.

Saunders R, Troje N (2011) A test battery for assessing biological motion perception. J Vis 11:686.

Shallo-Hoffmann JA, Bronstein AM, JF A, Morland A, Gresty MA (1998) Vertical and horizontal motion perception in congenital nystagmus. Neuro-Ophthalmology 19:171-183.
